# The novel protein DELAYED PALE-GREENING1 is required for early chloroplast biogenesis in *Arabidopsis thaliana*

**DOI:** 10.1038/srep25742

**Published:** 2016-05-10

**Authors:** Dong Liu, Weichun Li, Jianfeng Cheng

**Affiliations:** 1 College of Agronomy/Key Laboratory of Crop Physiology, Ecology and Genetic Breeding, Ministry of Education, Jiangxi Agricultural University, Nanchang, 330045, China

## Abstract

Chloroplast biogenesis is one of the most important subjects in plant biology. In this study, an Arabidopsis early chloroplast biogenesis mutant with a delayed pale-greening phenotype (*dpg1*) was isolated from a T-DNA insertion mutant collection. Both cotyledons and true leaves of *dpg1* mutants were initially albino but gradually became pale green as the plant matured. Transmission electron microscopic observations revealed that the mutant displayed a delayed proplastid-to-chloroplast transition. Sequence and transcription analyses showed that *AtDPG1* encodes a putatively chloroplast-localized protein containing three predicted transmembrane helices and that its expression depends on both light and developmental status. GUS staining for *AtDPG1::GUS* transgenic lines showed that this gene was widely expressed throughout the plant and that higher expression levels were predominantly found in green tissues during the early stages of Arabidopsis seedling development. Furthermore, quantitative real-time RT-PCR analyses revealed that a number of chloroplast- and nuclear-encoded genes involved in chlorophyll biosynthesis, photosynthesis and chloroplast development were substantially down-regulated in the *dpg1* mutant. These data indicate that AtDPG1 plays an essential role in early chloroplast biogenesis, and its absence triggers chloroplast-to-nucleus retrograde signalling, which ultimately down-regulates the expression of nuclear genes encoding chloroplast-localized proteins.

The chloroplast is an essential organelle in plant cells and plays important roles in primary metabolism, such as CO_2_ fixation, manufacture of carbon skeletons and fatty acids, and synthesis of amino acids from inorganic nitrogen^1^. The products of such metabolism are very useful not only for the plant itself but also for most living organisms.

Chloroplasts are the product of serial endosymbiotic events and arose from a cyanobacterial ancestor that was engulfed by a eukaryote^2^,^3^. In higher plants, the biogenesis of chloroplasts is initiated from undifferentiated, non-photosynthetic proplastids in shoot meristems. As shoot meristematic cells begin to differentiate into mesophyll cells, proplastids coordinately differentiate into chloroplasts^4^,^5^. Although the chloroplasts in true leaves develop from meristematic proplastids as the leaf primordia emerge, chloroplasts in cotyledons develop from etioplasts that are already present in mesophyll tissue within the embryo^6^. During photomorphogenesis, light is an important environmental cue triggering chloroplast biogenesis, a complex process in which photosynthetic pigments biosynthesis, the import of nuclear-encoded proteins, and the building of thylakoid networks embedded with photosynthetic electron transport complexes are integrated to establish fully functional chloroplasts^7^,^8^.

The biogenesis of chloroplasts requires the coordinated expression and assembly of proteins encoded by both the nuclear and chloroplast genomes. Most of the chloroplast proteins are encoded by nuclear genes, translated in the cytosol as precursor proteins, and post-translationally imported into the chloroplast stroma^9^. From the stroma, some intermediate precursors are translocated into or across the thylakoid membrane^10^. Because the majority of the several thousands of chloroplast proteins are encoded by the nucleus, it is not surprising that numerous mutants that are disrupted in chloroplast biogenesis have mutations on nuclear-encoded genes with diverse biological functions, including biosynthesis of photosynthetic pigments, thylakoid biogenesis, lipid biosynthesis, protein import, photosystem assembly, protein maturation and degradation and plastid gene expression^11^,^12^,^13^,^14^,^15^,^16^,^17^,^18^. Therefore, chloroplast biogenesis is a complex process in higher plants, and identifying chloroplast biogenesis mutants and illuminating their molecular mechanisms will provide significant insight into the complex process of chloroplast biogenesis.

The coordinated expression of proteins encoded by the chloroplasts and of those encoded by the nucleus but destined for the chloroplasts is assumed to be regulated by anterograde and retrograde communication between the nucleus and the chloroplasts^19^. Anterograde mechanisms coordinate gene expression in chloroplasts in response to endogenous and environmental signals that are perceived by the nucleus. Retrograde mechanisms transmit signals that originate in the chloroplasts to regulate nuclear gene expression^20^. In recent decades, significant advances have occurred in understanding the complexity of chloroplast-to-nucleus retrograde signalling. For example, norflurazon (NF), an inhibitor of carotenoid biosynthesis, causes the photooxidative destruction of chloroplasts and leads to a markedly decreased expression of a large number of nuclear photosynthesis-related genes^21^,^22^. Changes in plastid gene expression (PGE) and the photosynthetic electron transport chain (PET) also trigger chloroplast-to-nucleus retrograde signalling^19^,^21^,^23^. Several recent studies have investigated the role of retrograde signalling in regulating the expression of nuclear genes during several stresses, including high light, drought, low temperature, heat, and excess ammonium^24^,^25^,^26^,^27^,^28^,^29^. Therefore, the disruption of the functional and developmental state of the chloroplast by endogenous factors or exogenous environmental cues generates a wide variety of chloroplast retrograde signals that regulate the expression of many nuclear genes encoding chloroplast-localized proteins.

To identify new genes required for the early steps of chloroplast biogenesis, we isolated several (22 lines) T-DNA insertion *emb* (embryo defective) mutants from the Arabidopsis Biological Resource Center and focused our subsequent analysis on those mutants exhibiting very low levels of chlorophyll. This report describes a mutant named delayed pale greening1 (*dpg1*), in which chloroplast biogenesis is clearly compromised and in which the transcription of chloroplast- and nuclear-encoded genes involved in chlorophyll biosynthesis, photosynthesis and chloroplast development is repressed. Our results indicate that AtDPG1 plays an essential role in early chloroplast biogenesis, its absence triggers chloroplast-to-nucleus retrograde signalling, ultimately down-regulating the expression of the nuclear genes encoding chloroplast-localized proteins.

## Results

### Identification of a new delayed pale-greening1 (*dpg1*) mutant in *Arabidopsis thaliana*

**Figure 1:**
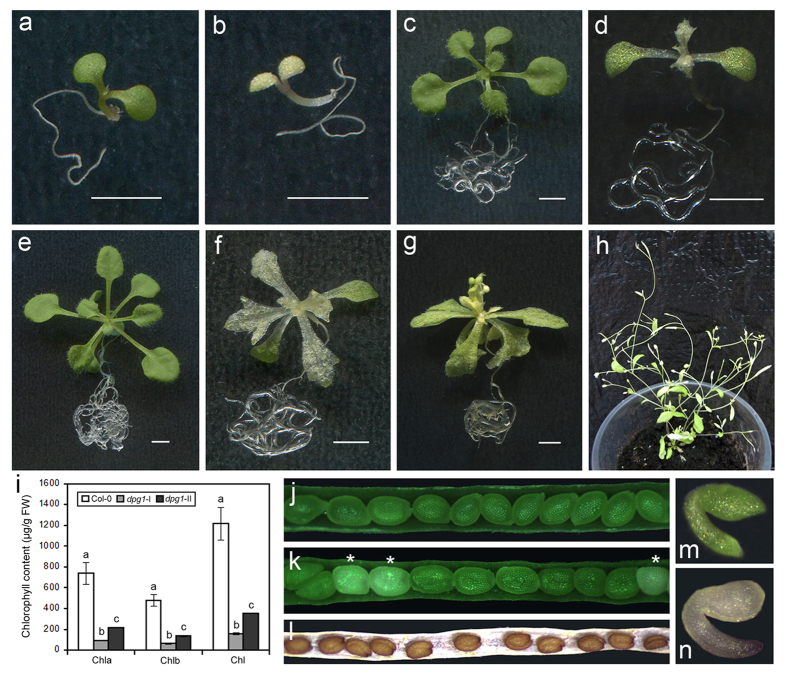
Phenotypic analysis of the *dpg1* mutant plants. (**a**) 5-day-old wild type. (**b**) 5-day-old *dpg1*. (**c**) 14-day-old wild type. (**d**) 14-day-old *dpg1*. (**e**) 21-day-old wild type. (**f**) 21-day-old *dpg1*. (**g**) 35-day-old *dpg1*. (**h**) 90-day-old *dpg1*. Scale bar = 5 mm. (**i**) The chlorophyll contents in the leaves of 21-day-old wild-type (Col-0), 21-day-old *dpg1* (*dpg1*-I) and 35-day-old *dpg1* (*dpg1*-II) plants. Chla, chlorophyll a; Chlb, chlorophyll b; Chl, total chlorophyll. Each value is the mean ± SE of three independent determinations, and different letters indicate significant differences at P < 0.05. (**j**) Green seeds in a 9-day-old wild-type silique. (**k**) Intermixed albino and green seeds in a 9-day-old *dpg1* heterozygous silique. The asterisks indicate albino seeds. (**l**) Seeds in a 15-day-old *dpg1* heterozygous silique. (**m**) A representative green embryo in a 9-day-old wild-type silique. (**n**) A representative albino embryo in a 9-day-old *dpg1* heterozygous silique.

To identify the genes involved in early chloroplast biogenesis, a delayed pale-greening mutant, designated *dpg1*, was isolated from the Arabidopsis Biological Resource Center. The homozygous mutant was completely albino and unable to grow on solid medium lacking a supplemental carbon source (Supplementary Fig. S1). However, this mutant survived and continued to grow on 1/2 MS medium containing 1% sucrose. While germinating normally, the mutant plants had albino cotyledons that became pale green several days later (Fig. 1a–d). In later developmental stages, the true leaves of the *dpg1* homozygous mutant were initially albino but gradually became pale green as the plant matured (Fig. 1c–g). When *dpg1* plants were grown for 5 weeks on 1/2 MS medium containing 1% sucrose and then transferred to soil, some of them withered and died because they were unable to resist the dry stress. The surviving *dpg1* plants looked weak and small, and their fertility was significantly reduced (Fig. 1h, Supplementary Table S1). At all growth stages, the mutant plants were greatly retarded in development, and they displayed a severe dwarf phenotype with small rosette leaves (Fig. 1a–h). Consistently with the phenotypes of *dpg1* homozygous mutant plants, the contents of chlorophyll a, chlorophyll b, and total chlorophyll in *dpg1* albino leaves were drastically lower than those in the wild type. After pale-greening, the contents of these pigments increased but were lower than those of the wild type (Fig. 1i). Additionally, within the immature siliques of the heterozygous *dpg1* mutant, abnormal albino seeds that were randomly distributed along the length of the siliques were detected at a frequency of approximately 25% (Fig. 1j,k,m,n, Table 1). The segregation ratios suggested that the seed phenotype is associated with one recessive mutation and that the albino seeds represent *dpg1* homozygous mutants. However, the seed colour was indistinguishable between the mutant and the wild type after seed desiccation (Fig. 1l).

### Molecular characterization and complementation of *dpg1*

**Figure 2:**
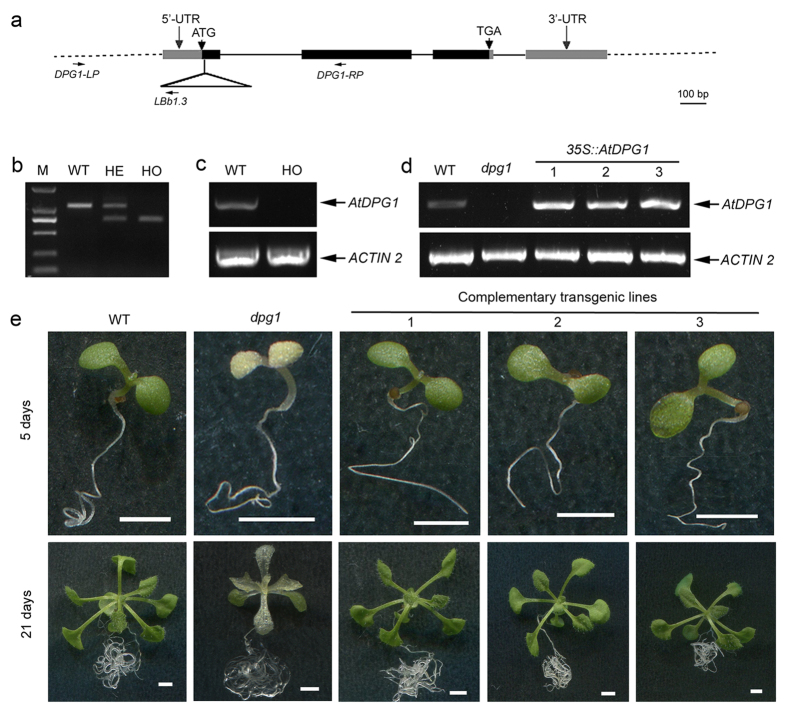
Molecular identification and phenotype rescue of the *dpg1* mutant. (**a**) Gene structure of At1g49510 and the position of the T-DNA insertion. Exons and introns are represented by boxes and lines, respectively. The insertion site of the T-DNA in *dpg1* is indicated by a triangle. *DPG1-LP*, *DPG1-RP* and *LBb1.3* are the primers that were used for genotyping PCR. (**b**) A representative genotyping PCR result of *dpg1*. Offspring of the heterozygous *dpg1* were segregated into three genotypes: wild type (WT), heterozygous (HE) and homozygous (HO). (**c**) Semi-quantitative RT-PCR analysis of *AtDPG1* in the wild type (WT) and homozygous *dpg1* mutant (HO). The *ACTIN2* gene was used as an internal control. (**d**) Semi-quantitative RT-PCR analysis of the *AtDPG1* transcription levels in the wild type (WT), the homozygous *dpg1* and three complementary transgenic lines (Lines 1, 2 and 3). The *ACTIN2* gene was used as an internal control. (**e**) The phenotype of a *dpg1* homozygous plant was rescued by transformation with a *35S::AtDPG1* transgene. Scale bar = 2 mm.

To explore the nature of the mutation, a heterozygous *dpg1* mutant line (female parent) was backcrossed with the wild type (male parent). The F_1_ progeny displayed a normal green phenotype, suggesting that the mutation was recessive (data not shown). To determine whether the T-DNA insertion co-segregates with the delayed pale-greening phenotype, DNA from F_2_ progenies was used as a template for PCR analysis. Primers were designed to amplify a larger fragment from wild-type plants and a smaller fragment from homozygous mutants. In heterozygous mutants, both of the fragments were amplified (Fig. 2b), suggesting that the T-DNA insertion co-segregated with the mutant phenotype. Sequencing the T-DNA left border junction indicated that the T-DNA was inserted into the first exon of At1g49510 (Fig. 2a). Consistently with this result, no transcript of At1g49510 was detected in the homozygous lines (Fig. 2c). These results indicate that At1g49510 is a candidate gene for *AtDPG1*.

To further confirm that the *AtDPG1* gene was At1g49510, we performed a complementation experiment. A 723-bp wild-type coding sequence (CDS) driven by the cauliflower mosaic virus 35S (*CaMV* 35S) promoter was introduced into *dpg1* heterozygous plants. Then, 19 T_1_ transgenic plants were screened out. Among these plants, 8 were *DPG1/dpg1* heterozygotes. The segregation rates of the offspring of these plants were far from 3:1 (green plants: albino plants; Supplementary Table S3). Furthermore, we determined the genotype of these offspring (T_2_ generation) and obtained *dpg1/dpg1* homozygous plants from 3 T_1_ transgenic lines that carried fragments of the exogenous coding sequence. These plants displayed green cotyledons and true leaves after their germination (Fig. 2e). A control transformation of the wild-type with the *35S::AtDPG1* transgene further confirmed that the rescue was complete and that overexpression did not produce significant phenotypic changes (Supplementary Fig. S2). A semi-quantitative RT-PCR analysis also showed that the transcripts of At1g49510 were present in these plants (Fig. 2d). These results indicated that the 723-bp CDS fragment can successfully complement the mutated phenotype and that the *AtDPG1* gene is At1g49510.

### Chloroplast development in *dpg1*

**Figure 3:**
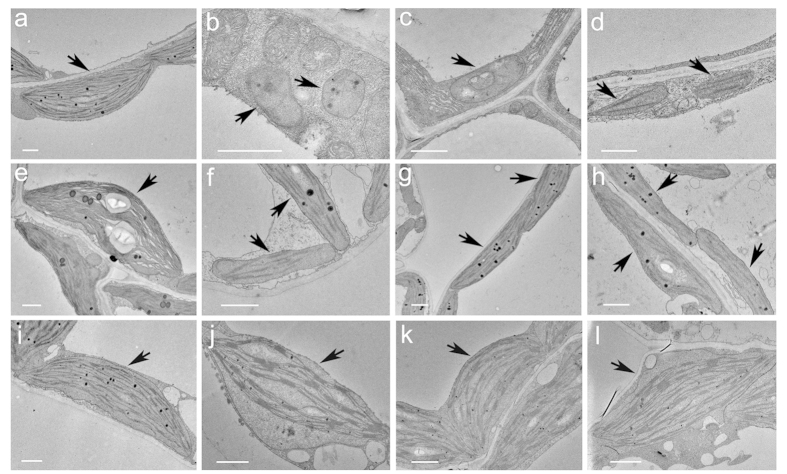
Ultrastructure of chloroplasts from wild-type, *dpg1* and complementary plant leaves. (**a–d**) Chloroplast structures from 21-day-old wild-type (**a**) and *dpg1* (**b–d**) plants. (**e**–**h**) Chloroplast structures from 35-day-old wild-type (**e**) and *dpg1* (**f–h**) plants. (**i–l**) Chloroplast structures from 21-day-old wild-type (**i**) and complementary (**j–l**) plants. The arrowheads indicate chloroplasts in the wild type, *dpg1* and complementary plants. Scale bar = 1 μm.

To examine the developmental status of the chloroplast in a *dpg1* mutant, mesophyll cell plastids from 21- and 35-day-old mutant plants (exhibiting albino and pale-green leaves, respectively) were analysed by transmission electron microscopy. In wild-type plants, chloroplasts were crescent-shaped and contained well-developed thylakoid membranes with large grana stacks (Fig. 3a,e). However, the albino leaves of 21-day-old *dpg1* seedlings contained many abnormal chloroplasts (Fig. 3b–d). According to their morphologies, these abnormal chloroplasts could be classified into three types. The first type was rounded, with almost no thylakoid membrane, and it appeared undifferentiated (Fig. 3b). The second type was oval-shaped, and no organized thylakoid membranes were observed except for a few circular internal membranes (Fig. 3c). The third type had very few but relatively continuous thylakoid membranes (Fig. 3d). The pale-green leaves of 35-day-old *dpg1* seedlings contained differentiated chloroplasts that were elongated and had fewer thylakoid membranes than did chloroplasts of wild-type seedlings at the same growth stage (Fig. 3f–h). These chloroplasts resembled wild-type chloroplasts at an early stage of development. Some of the thylakoid membranes were arranged as grana stacks. Moreover, the chloroplast morphology, size, thylakoid abundance, and grana stacking were restored to those of the wild type in *dpg1* plants expressing the *35S::AtDPG1* transgene (Fig. 3i–l). Altogether, these observations indicated that the delayed transition of proplastids to chloroplasts in *dpg1* is consistent with the delayed pale-greening phenotype.

### *AtDPG1* encodes a putatively chloroplast-localized protein that is highly conserved in various dicots

**Figure 4:**
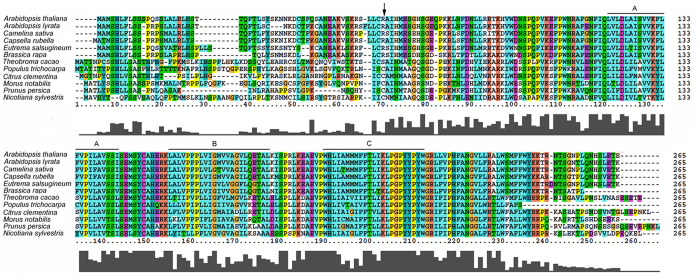
Multiple sequence alignment of AtDPG1 and its homologues. The transmembrane domains (**A–C**) that were conserved among the DPG1 homologues are indicated. Protein sequences of DPG1 homologues from *Arabidopsis lyrata* (XP_002891514), *Camelina sativa* (XP_010479384), *Capsella rubella* (XP_006304157), *Eutrema salsugineum* (XP_006393250), *Brassica rapa* (XP_009124376), *Theobroma cacao* (XP_007042342), *Populus trichocarpa* (XP_006384460), *Citrus clementina* (XP_006423083), *Morus notabilis* (XP_010096165), *Prunus persica* (XP_007201809), and *Nicotiana sylvestris* (XP_009792237) were retrieved from GenBank. The downward arrow indicates the putative cleavage site of the transit peptide of AtDPG1 as predicted by ChloroP 1.1 program. Sequences were aligned with ClustalX 2.0 software.

BLAST searches of the complete *Arabidopsis thaliana* sequence revealed that only one copy of the *AtDPG1* gene is present in the nuclear genome, and it encodes a 240-amino acid protein with an apparent molecular mass of 27.38 kD and a theoretical pI of 10.27. The ChloroP 1.1, Predotar and iPSORT programs predicted that the AtDPG1 protein is targeted to the chloroplast and that its 55- amino-acid N-terminal region is likely to be a transit peptide (Fig. 4, Supplementary Table S4); this result was also reported in the SubCellular Database (http://suba.plantenergy.uwa.edu.au/flatfile.php?id). Consistently with this result, proteomic data have also indicated that AtDPG1 is most likely localized to the chloroplast envelope^30^. Moreover, trans-membrane helix prediction through TMHMM indicated the presence of 3 trans-membrane helices in AtDPG1 (Fig. 4, Supplementary Fig. S3). A search of the GenBank database (http://blast.ncbi.nlm.nih.gov/Blast.cgi) revealed dozens of ESTs that are highly homologous to AtDPG1 in diversified species of dicots but not in monocots (Fig. 4). AtDPG1 is 93%, 89%, 87%, 83%, 79%, 66%, 63%, 59%, 55%, 53%, and 52% identical to the putative DPG1s of *Arabidopsis lyrata* (AlDPG1), *Camelina sativa* (CsDPG1), *Capsella rubella* (CrDPG1), *Eutrema salsugineum* (EsDPG1), *Brassica rapa* (BrDPG1), *Populus trichocarpa* (PtDPG1), *Citrus clementina* (CcDPG1), *Prunus persica* (PpDPG1), *Theobroma cacao* (TcDPG1), *Morus notabilis* (MnDPG1), and *Nicotiana sylvestris* (NsDPG1), respectively. To investigate the evolutionary relationship among DPG1 homologues, a phylogenetic analysis was performed. As shown in Supplementary Fig. S4, the proteins from Brassicaceae species and other dicots clustered in different subclades. Although there was a robust separation within these two big clades, some bootstrap percentages were low, thus making the analysis a less robust interpretation within Brassicaceae species and other dicots.

### Promoter activity analysis of the *AtDPG1* gene

**Figure 5:**
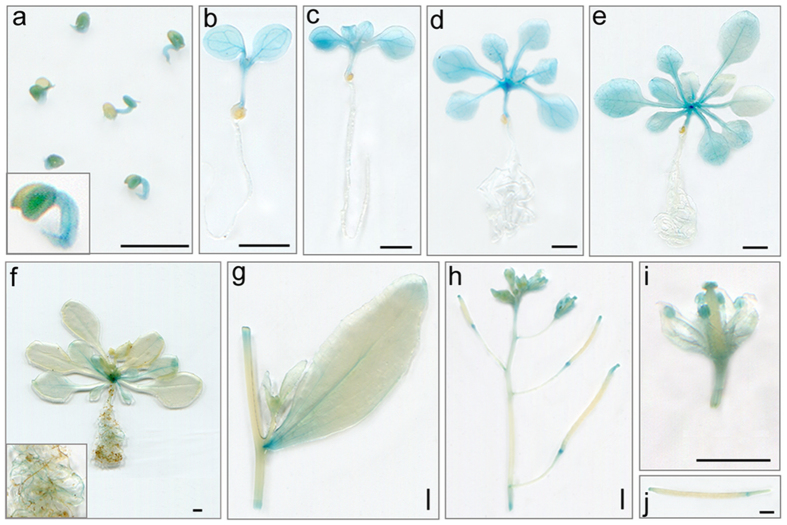
Histochemical assays for the expression pattern of *AtDPG1::GUS* transgenic lines as visualized by GUS staining. (**a**) Germinating seeds (36 h after sowing). (**b**) 5-day-old seedling. (**c**) 7-day-old seedling. (**d**) 14-day-old plant. (**e**) 21-day-old plant. (**f**) 35-day-old plant. (**g**) Stem and cauline leaf. (**h**) Inflorescence. (**i**) Fully opened flower. (**j**) Silique. Scale bar = 2 mm.

To investigate the possible physiological function of the *AtDPG1* gene, the temporal and spatial expression patterns of the *GUS* gene driven by the *AtDPG1* promoter were analysed in transgenic Arabidopsis plants. A 1,811-bp promoter fragment of the *AtDPG1* gene was fused to the *GUS* reporter gene, and the expression cassette was introduced into wild-type Arabidopsis plants. According to the observed pattern, *AtDPG1* was expressed very early in germinating seeds (Fig. 5a). In young seedlings, strong *AtDPG1*-GUS-driven expression appeared in the cotyledons, newly emerging true leaves and hypocotyls, but no detectable expression was observed in the roots (Fig. 5b,c). In vegetative plants, GUS staining was also observed in the green tissues, including cotyledons, true leaves and hypocotyls, but not in the roots (Fig. 5d,e). At a later stage (extended to the bolting stage), GUS expression was detected at a low level in the cotyledons, true leaves, cauline leaves and stems (Fig. 5f,g). Moreover, very weak GUS staining was also detected in partial roots (Fig. 5f). When the transgenic plants entered the reproductive stage, stronger GUS expression was detected in the inflorescence (Fig. 5h). Further examination of GUS expression showed that this gene was mainly expressed in the stamens and stigmas of open flowers (Fig. 5i). In addition, weak GUS activity was also found at the base and the tip of silique (Fig. 5h,j). GUS staining for transgenic lines showed that *AtDPG1* was widely expressed throughout the plant and that higher expression levels were predominately found in green tissues during the early stages of Arabidopsis seedling development.

### Expression pattern of the *AtDPG1* gene

**Figure 6:**
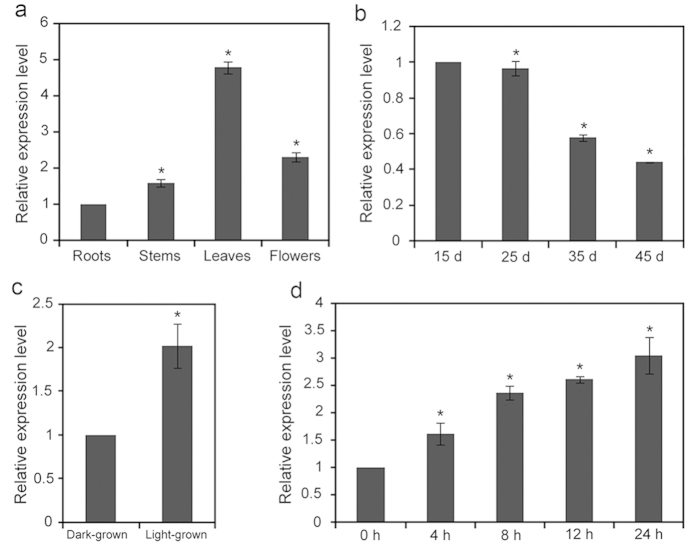
Expression pattern of the *AtDPG1* gene. (**a**) Quantitative real-time PCR analysis of *AtDPG1* transcripts in different organs (roots, stems, leaves, and flowers) from 45-day-old wild-type Arabidopsis plants. The expression levels of *AtDPG1* displayed a range of cycle C_t_ values ranging from 22.80 to 24.44 in different tissues. The roots expressed the lowest levels of *AtDPG1* mRNA, with a C_t_ value of 24.44, and the *AtDPG1* transcript level in roots was set to 1.0. (**b**) *AtDPG1* transcript levels in the leaves of different developmental stages in wild-type Arabidopsis plants. The *AtDPG1* transcript level in the leaves from 15-d-old seedlings was set to 1.0. (**c**) Transcript levels of *AtDPG1* in 4-day-old wild-type light-grown and dark-grown seedlings. The *AtDPG1* transcript level in the dark-grown seedlings was set to 1.0. (**d**) Transcript levels of *AtDPG1* during the light-induced greening of etiolated wild-type seedlings. After growth in darkness for 4 days, the etiolated Arabidopsis seedlings were illuminated for 4, 8, 12, and 24 h. The *AtDPG1* transcript level in the etiolated seedlings was set to 1.0. The bar graphs show the relative expression levels, as determined by the comparative C_T_ method and normalized to the expression of the *ACTIN2* gene. The error bars indicate the interval delimited by 2^−(ΔΔCT ± SD)^. The asterisks indicate ΔC_T_ values significantly different from those of the control in a Mann–Whitney U-test (P < 0.05).

To elucidate the possible role of *AtDPG1* in the course of plant growth and development, we examined its organ-specific expression. As shown in Fig. 6a, the leaves expressed the highest levels of *AtDPG1* mRNA, whereas these levels in other organs of Arabidopsis plants were significantly lower. The roots accumulated the lowest level of all of the analysed tissues. To study the expression of *AtDPG1* at different developmental stages, the levels of the *AtDPG1* transcript in leaves ranging from 15 to 45 days old were evaluated. The expression level of *AtDPG1* decreased as the age of the leaves increased. The *AtDPG1* transcript content of leaves of 45-day-old plants was only approximately 40% of that detected in leaves of 15-day-old plants (Fig. 6b).

Light induces the differentiation of non-photosynthetic proplastids into fully functional chloroplasts. To examine the effects of light on the expression of *AtDPG1*, we used real-time PCR to determine *AtDPG1* expression in 4-d-old wild-type etiolated seedlings exposed to light for 4, 8, 12 and 24 h. As shown in Fig. 6c, the *AtDPG1* transcript levels were high under light and much lower in the dark in 4-d-old etiolated seedlings. Furthermore, *AtDPG1* expression was highly induced after illumination for 4 h and peaked after 24 h of illumination (Fig. 6d). These observations suggest that light plays a major role in regulating *AtDPG1* expression.

### Effects of the *AtDPG1* mutation on the expression of chloroplast-encoded genes

**Figure 7:**
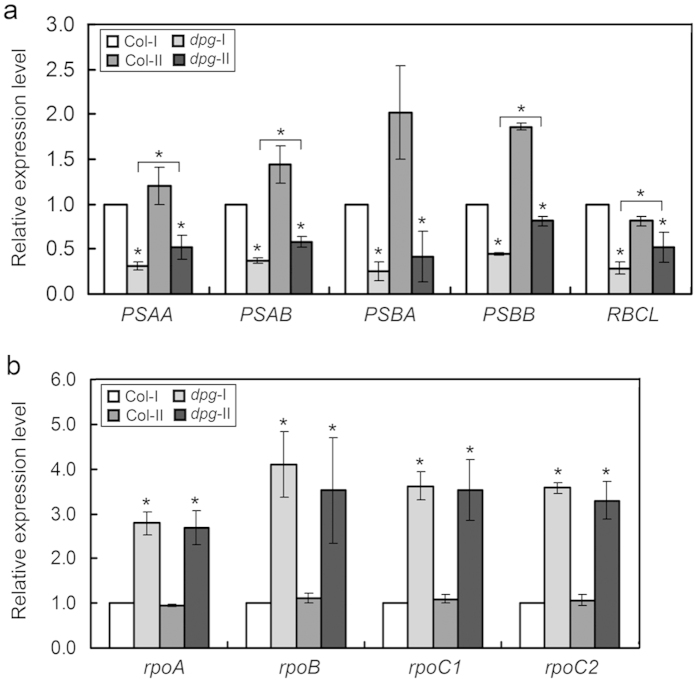
Effects of the *AtDPG1* mutation on the expression of chloroplast-encoded genes. (**a**) The expression levels of chloroplast-encoded PEP-dependent genes (*PSAA*, *PSAB*, *PSBA*, *PSBB*, and *RBCL*). (**b**) The expression levels of genes encoding PEP core subunits (*rpoA*, *rpoB*, *rpoC1*, and *rpoC2*). The terms *dpg1*-I and *dpg1*-II refer to the leaves that were albino or pale-green in 21-day-old and 35-day-old *dpg1* mutants, respectively. The transcript level of each gene was set to 1.0 in the leaves from a 21-day-old wild type plant. The bar graphs show the relative expression levels, as determined by the comparative C_T_ method and normalized to the expression of the *ACTIN2* gene. Error bars indicate the interval delimited by 2^−(ΔΔCT ± SD)^. The asterisks indicate ΔC_T_ values significantly different from those of the control in a Mann–Whitney U-test (P < 0.05).

The expression of chloroplast-encoded genes is tightly associated with chloroplast developmental status. Because abnormal chloroplast development was observed in *dpg1*, we compared the transcript levels of various chloroplast-encoded genes between wild-type plants and *dpg1* mutants. The five genes *PSAA* (encoding the PSAA protein, comprising the reaction centre for photosystem I), *PSAB* (encoding the D1 subunit of the photosystem I reaction centre), *PSBA* (encoding the D1 subunit of the photosystem II reaction centre core), *PSBB* (encoding the CP47 subunit of the photosystem II reaction centre), and *RBCL* (encoding the large subunit of Rubisco) were selected as plastid-encoded polymerase (PEP)-dependent genes. The expression levels of these genes were markedly lower in the mutant than in the wild type; however, the expression levels of most of these genes (*PSAA*, *PSAB*, *PSBB*, and *RBCL*) mildly increased as the albino leaves became pale green (Fig. 7a). Because the albino phenotype of *dpg1* mutant was similar to that of mutants with a defect in PEP function^31^,^32^,^33^,^34^, *dpg1* is probably a PEP-defective mutant. PEP is composed of plastid-encoded core subunits and a multiple nuclear-encoded sigma factor that confers promoter specificity to PEP. The core subunits of PEP are encoded by the plastid *rpoA*, *rpoB*, *rpoC1*, and *rpoC2* genes, which are nucleus-encoded polymerase (NEP)-dependent genes. In the *dpg1* mutant, the transcript levels of all four genes were several times greater than those of the wild type (Fig. 7b).

### Effects of the *AtDPG1* mutation on the expression of nuclear-encoded photosynthetic genes

**Figure 8:**
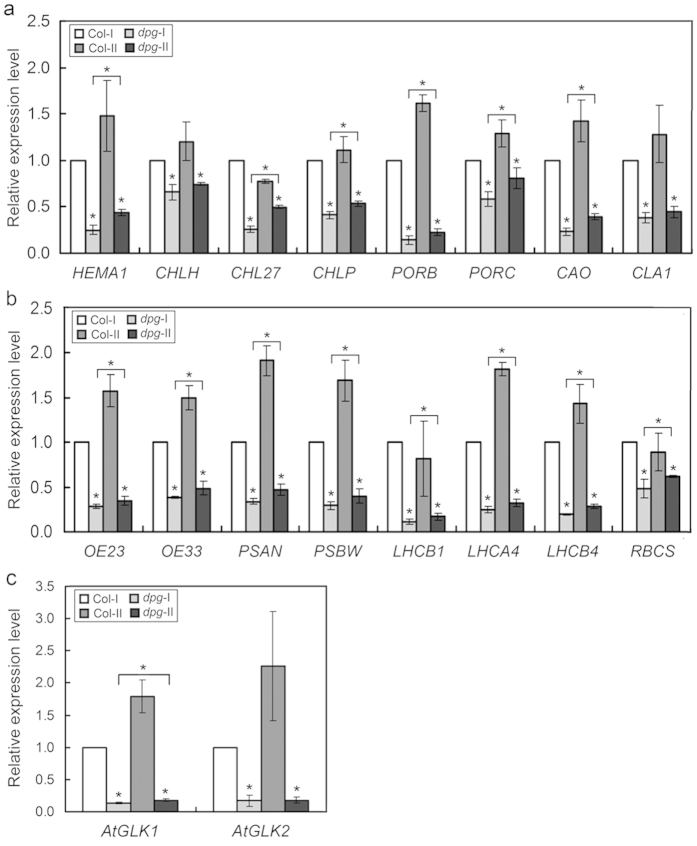
Effects of the *AtDPG1* mutation on the expression of nuclear-encoded photosynthetic genes. (**a**) The expression levels of nuclear-encoded genes that are critical for chlorophyll biosynthesis (*HEMA1*, *CHLH*, *CHL27*, *CHLP*, *PORB*, *PORC*, *CAO*, and *CLA1*). (**b**) The expression levels of nuclear-encoded photosynthetic genes (*OE23*, *OE33*, *PSAN*, *PSBW*, *LHCB1*, *LHCA4*, *LHCB4*, and *RBCS*). (**c**) The expression levels of nuclear-encoded GLK transcription factor genes (*AtGLK1* and *AtGLK2*). The terms *dpg1*-I and *dpg1*-II refer to the leaves that were albino or pale-green in 21-day-old and 35-day-old *dpg1* mutants, respectively. The transcript level of each gene was set to 1.0 in the leaves from a 21-d-old wild type plant. The bar graphs show the relative expression levels, as determined by the comparative C_T_ method and normalized with the expression of the *ACTIN2* gene. The error bars indicate the interval delimited by 2^–(ΔΔCT ± SD)^. The asterisks indicate ΔC_T_ values significantly different from those of control in a Mann–Whitney U-test (P < 0.05).

The developmental and functional status of chloroplasts controls the transcription of nuclear-encoded genes via retrograde signaling^19^,^35^. We next examined the transcription of nuclear-encoded genes associated with chlorophyll biosynthesis and photosynthesis in the *dpg1* mutant. The transcription of nuclear-encoded genes that are critical for chlorophyll biosynthesis was first analysed, including genes encoding glutamyl-tRNA reductase (HEMA1), magnesium chelatase (CHLH), Mg-protoporphyrin IX monomethyl ester cyclase (CHL27), geranylgeranyl reductase (CHLP), protochlorophyllide oxidoreductase B (PORB), protochlorophyllide oxidoreductase C (PORC), chlorophyllide a oxygenase (CAO), and 1-deoxy-D-xylulose 5-phosphate synthase 1 (CLA1). These genes were down-regulated in the *dpg1* mutant compared with the wild type; however, the expression levels of most of these genes (*HEMA1*, *CHL27*, *CHLP*, *PORB*, *PORC*, and *CAO*) modestly increased as the albino leaves became pale green (Fig. 8a). The transcription of nuclear-encoded photosynthetic genes was next examined, including genes encoding the proteins of the oxygen-evolving complex of photosystem II (OE23 and OE33), the N subunit of photosystem I (PSAN), the PSBW protein similar to the photosystem II reaction centre subunit W, the chlorophyll a/b binding proteins (LHCB1, LHCA4, and LHCB4), and the rubisco small subunit (RBCS). Similarly, the transcription levels of all eight genes in the *dpg1* mutant were significantly lower than that in the wild-type plant; however, the expression levels of these genes mildly increased as the albino leaves became pale green (Fig. 8b). Previously, the *GOLDEN2-LIKE* (GLK) transcription factors AtGLK1 and AtGLK2 were identified as positive regulators of nuclear-encoded photosynthetic genes by binding to their promoter sequences^8^. Further analyses of the *AtGLK1* and *AtGLK2* transcript levels revealed that the two genes were significantly down-regulated in the *dpg1* mutant compared with the wild type; however, the expression levels of the *AtGLK1* gene modestly increased as the albino leaves became pale green (Fig. 8c).

## Discussion

Chloroplasts contain several thousand different proteins, of which more than 95% are encoded by nuclear genes, synthesized in the cytosol as precursor proteins, and imported into the organelle^36^. Despite the discovery of a large fraction of genes involved in chloroplast development^37^, full understanding of the complex biogenesis of this organelle is lacking. In this study, the novel nuclear gene *AtDPG1* was identified using a loss-of-function forward genetic approach. Although AtDPG1 has previously been isolated as EMBRYO DEFECTIVE 1273 (EMB1273) by Tzafrir *et al.*^38^, very limited information about the function of Arabidopsis AtDPG1 proteins is available. We first searched for members of this gene family in the GenBank database. The results revealed that the DPG1 protein is highly conserved in various dicots but not in monocots (Fig. 4), suggesting that the origination of *DPG1* might have occurred after the separation of monocots and dicots. Consistently with the results of the sequence alignment, the iPSORT, ProteinProwler and ChloroP programs also yielded a high probability for chloroplast localization for DPG1 in higher plants (Supplementary Table S4). Proteomic data have also indicated that AtDPG1 is most likely localized to the chloroplast envelope^30^. Despite this evidence, the chloroplast localization of AtDPG1 needs to be experimentally verified in further investigations.

In dicotyledonous plants, the development of chloroplasts differs between cotyledons that initially act primarily as storage tissue and true leaves whose major function is photosynthesis. In cotyledons, plastids partially develop during embryogenesis, but this development arrests during seed maturation. When seeds germinate in the light, the plastids further develop into functional chloroplasts. In contrast to chloroplast development in cotyledons, in true leaves, chloroplasts develop directly from the proplastids in the shoot apex^5^,^39^. These differences have been detected in several mutants with chloroplast defects restricted either to cotyledons or to true leaves. For instance, the *sco* mutant group and the *cyo1* and *wco* mutants have albino cotyledons but normal green true leaves^40^,^41^,^42^,^43^. In contrast, in the *var2* and *sg1* mutants, Arabidopsis cotyledons develop normally, but true leaves contain chlorophyll-deficient white sectors^44^,^45^. In our study, both the cotyledons and true leaves of *dpg1* were initially albino and then gradually turned pale-green during development in *dpg1* plants, although abnormal white embryos were observed during the early stages of embryogenesis (Fig. 1), suggesting that AtDPG1 plays an equally important role in early chloroplast development in the cotyledons and true leaves of Arabidopsis seedlings. A multiple-sequence alignment analysis showed that DPG1 is conserved in dicots but not in monocots. Given that the chloroplasts of monocots and dicots are highly similar except for some metabolic issues, DPG1 may play a specific role in chloroplast metabolism in dicots. The most striking feature of *dpg1* was the delayed pale-greening of both its cotyledons and true leaves (Fig. 1). Furthermore, ultrastructural findings demonstrated that thylakoid membranes in the young leaves of *dpg1* mutants were less abundant than in wild type, but their abundance significantly increased as the leaves matured (Fig. 3). GUS staining for *AtDPG1::GUS* transgenic lines showed that promoter of *AtDPG1* is active in green tissues but not in roots during the early stages of Arabidopsis seedling development (Fig. 5b–e). Quantitative real-time RT-PCR analysis also revealed that *AtDPG1* was expressed in leaves in a light-inducible way (Fig. 6c,d). Light is one of the most crucial environmental factors stimulating the differentiation of non-photosynthetic proplastids into photosynthetic chloroplasts. The increased accumulation of the *AtDPG1* transcript after light illumination suggests that this gene may play an important role in the light-regulated chloroplast biogenesis. Our results also showed that the transcripts of both chloroplast- and nuclear-encoded photosynthetic genes were severely suppressed during the early stage in the *dpg1* mutant; however, the expression levels of most of the genes mildly increased as the albino leaves became pale green (Figs 7a and 8). Altogether, these findings show that AtDPG1 plays an essential role in early stages of chloroplast biogenesis and that functional chloroplast biogenesis depends on leaves reaching a more mature developmental stage in the *dpg1* mutant.

The young leaves of *dpg1* mutants were initially albino but gradually turned to pale-green as the plant matured. There are two possible explanations for the seedling-stage-specific albino phenotype. The first explanation is that other proteins may partly compensate for the absence of AtDPG1 during later development stages. For example, the homozygous *sig6-1* mutant plants had pale green cotyledons with drastically reduced chlorophyll content in 3-day-old Arabidopsis seedlings. However, the chlorophyll deficiency is limited to the young cotyledons and is restored to the wild-type level in 8-day-old seedlings^46^. Further studies have revealed that another late general sigma factor (possibly AtSIG1) is likely to have overlapping functions with AtSIG6 to compensate for its deficiency in *sig6-1* mutants when 8 or more days old^46^,^47^. The second explanation is that AtDPG1 is not required for later developmental stages of chloroplast development in Arabidopsis. Quantitative real-time PCR analysis revealed that the expression level of *AtDPG1* decreased as the age and developmental state of the leaves increased. The *AtDPG1* transcript content of leaves in 45-day-old plants was only approximately 40% of that in 15-day-old plants (Fig. 6b). Although the leaves of 45-day-old plants displayed an approximately 5-fold increase in relative expression compared with that in root (Fig. 6a), there was a low absolute expression of *AtDPG1,* because GUS staining showed very weak expression of *AtDPG1* in the roots (Fig. 5f). These results suggest that AtDPG1 plays specific roles in early stages of chloroplast development. The phenotype of *dpg1* is similar but not identical to the formerly described mutant phenotype, which is characterized by young albino leaves with drastically reduced chlorophyll but nearly normal levels of chlorophyll in mature leaves. For instance, the Arabidopsis *dg1* and *ys1* mutants display a seedling-stage-specific albino or yellow seedling phenotype caused by delayed chloroplast development. Further studies have revealed that *DG1* encodes a chloroplast-targeted PPR protein and that *YS1* encodes a DYW protein that is required to edit *rpoB* transcripts^32^,^48^. Despite this evidence, only a few genes that are responsible for the seedling-stage-specific phenotype have been isolated, and their molecular mechanisms remain elusive. Therefore, elucidating the complex molecular mechanisms regulating chloroplast biogenesis in higher plants remains a major task for future research.

An analysis of chloroplast gene expression revealed that the transcript levels of PEP-dependent genes were significantly reduced in the *dpg1* mutant (Fig. 7a), suggesting that the *dpg1* mutant is severely impaired in PEP activity and that AtDPG1 may play an important role in the regulation of chloroplast gene expression. However, the results of expression analysis showed that the genes encoding PEP core subunits were dramatically induced in *dpg1* (Fig. 7b). The increased transcript abundance of these genes may indicate a feedback mechanism to increase PEP levels to transcribe PEP-dependent genes. Even so, the accumulation of transcripts for PEP components did not result in the formation of functional PEP, because the expression of PEP-dependent genes decreased in the *dpg1* mutant (Fig. 7a). A defect in polysome assembly has been shown to be accompanied by a decrease in the level of the *RBCL* mRNA^49^. In *dpg1* mutant, the expression of *RBCL* drastically decreased (Fig. 7a), indicating that a loss-of-function mutation in *AtDPG1* may affect the assembly of chloroplast ribosomes, eventually leading to the disruption of chloroplast translation during early chloroplast development.

The chloroplast developmental status controls a series of nuclear genes that encode chloroplast-localized proteins through retrograde signalling^19^,^35^,^50^,^51^. In the present study, we examined the transcription levels of nuclear-encoded genes that are associated with chlorophyll biosynthesis and photosynthesis in the *dpg1* mutant. Quantitative real-time PCR analysis showed that the transcripts of these genes were severely suppressed during the early stage in the *dpg1* mutant, but the expression levels of most of these genes mildly increased as the albino leaves became pale green (Fig. 8). The leaves of *dpg1* were initially albino and then gradually turned pale-green during development in *dpg1* plants (Fig. 1). Furthermore, ultrastructural observations showed that thylakoid membranes were less abundant in the albino leaves of mutant plants than in the wild type; however, their abundance gradually increased as the leaves became green (Fig. 3). It is likely that the retrograde signals derived from these impaired chloroplasts regulate the expression of nuclear genes encoding chloroplast-localized proteins. Several studies have shown that GOLDEN2-LIKE transcription factors (GLK) modulate chloroplast development^52^,^53^ and mediate retrograde signalling^8^,^54^ in response to the functional state of the chloroplast. The expression levels of *AtGLK1* and *AtGLK2* are tightly associated with the expression of nuclear photosynthesis genes in mutants that are defective in chloroplast structure and function^18^,^54^. Consistently with these findings, in the leaves of *dpg1* plants, both *AtGLKs* (*AtGLK1* and *AtGLK2*) had significantly reduced transcript levels (Fig. 8c). It is confirmed that chloroplast-to-nucleus retrograde signalling was evident in the *dpg1* mutant. Furthermore, because AtGLKs regulate the expression of the *LHCB* gene family, as well as *HEMA1*^8^,^53^, the decreased expression of these nuclear-encoded photosynthetic genes in the *dpg1* mutant may occur through the regulation of *AtGLKs* expression.

In summary, the novel nuclear gene *AtDPG1* was identified, and its loss-of-function mutation resulted in defects in chloroplast structure and function. The expression of several nuclear-encoded genes involved in chlorophyll biosynthesis, photosynthesis and chloroplast development was substantially down-regulated in the *dpg1* mutant, suggesting that chloroplast defects trigger a chloroplast-to-nucleus retrograde signal that may account for such transcriptional changes. Further studies of the biochemical properties of AtDPG1 will shed more light on the function of this protein in chloroplast biogenesis.

## Methods

### Plant material and growth conditions

*Arabidopsis thaliana* ecotype Columbia (Col-0) was used in all experiments. After 3 days of stratification in the dark at 4 °C, the surface-sterilized seeds were germinated on 1/2 MS medium supplemented with 1% (w/v) sucrose at 22 °C with a 16-h-light/8-h-dark cycle. The *dpg1* (SALK_004621C) T-DNA insertion line was obtained from the Arabidopsis Biological Resource Center^55^. The T-DNA insertion site was identified by sequencing PCR products that were amplified from the mutant with a T-DNA primer (*LBb1.3*) and the gene-specific primers *DPG1-LP* and *DPG1-RP*. All of the primers used are listed in Supplementary Table S2.

### Constructs and plant transformation

To construct the *PDPG1::GUS* fusion gene, a 1,811-bp DNA fragment upstream of the ATG start codon of the *AtDPG1* gene (At1g49510) was amplified from *Arabidopsis thaliana* genomic DNA by PCR. The pair of primers used in the PCR was *PDPG1-F* and *PDPG1-R* (*Hind* III and *Nco* I sites were introduced). The specific PCR fragment was then inserted into binary vector pCAMBIA1301 between *Hind* III and *Nco* I sites, replacing the *CaMV 35S* promoter, to create the recombinant transcription unit *PDPG1::GUS*. For the construction of *35S::DPG1* unit, the full-length coding sequence (CDS) corresponding to the *AtDPG1* gene (At1g49510) locus was cloned by using RT-PCR from *Arabidopsis thaliana*. The pair of primers used in the PCR was *OEDPG1-F* and *OEDPG1-R* (*Nco* I and *Bst*E II sites were introduced). The specific PCR fragment was then inserted into binary vector pCAMBIA1301 between *Nco* I and *Bst*E II sites, replacing the *GUS* gene, to create the recombinant transcription unit *35S::DPG1*. The recombinant plasmids were then introduced into *Agrobacterium tumefaciens* strain GV3101 and transformed into wild-type Arabidopsis or heterozygous *dpg1* mutant plants using the floral dip method^56^. The transformants were then screened on 1/2 MS medium containing 50 μg ml^−1^ hygromycin. All primers used are listed in Supplementary Table S2.

### RNA extraction, cDNA synthesis, and gene expression analysis

Total RNA was extracted from plant tissues with TRI Reagents, this was followed by treatment with RNase-free DNase I (TaKaRa, Dalian, China) to degrade genomic DNA. First-strand cDNA was synthesized from 2.0 μg of total RNA using ImProm-II^TM^ reverse transcriptase (Promega, Madison, WI, USA) in a 10.0-μl reaction following the manufacturer’s instructions with minor modifications. For semi-quantitative RT-PCR analysis, PCR mixes were subjected to 34 cycles for the *AtDPG1* gene and 28 cycles for the *ACTIN2* gene. Three independent biological repeats were used to generate the representative results shown here. For quantitative real-time PCR analysis, the reaction was performed using SYBR Green Perfect mix (TaKaRa, Dalian, China) on a CFX96 (Bio-Rad) following the manufacturer’s instructions. Gene expression was normalized to that of *ACTIN2* by subtracting the C_T_ value of *ACTIN2* from the C_T_ value of the gene of interest. Expression ratios were then obtained from the equation 2^−ΔΔCT^. For each sample, quantitative real-time PCR was performed with three technical replicates from three biological replicate samples. Efficiency-corrected ΔC_T_ values were calculated, and ΔΔC_T_ was used to quantify the relative differences in transcript accumulation. The primers for the genes of interest are listed in Supplementary Table S2.

### Transmission electron microscopy (TEM) analysis

The leaves of Arabidopsis were fixed in 2.5% (w/v) glutaraldehyde in phosphate buffer (pH 7.4) overnight at 4 °C. Thereafter, the samples were rinsed thoroughly with the same buffer 3-6 times and post-fixed with 1% (w/v) osmium tetroxide for 2 hours at 4 °C. Then the samples were dehydrated in a graded ethanol series (v/v, 30%, 50%, 70%, 90%) and in 1:1 mixture of 90% ethanol and 90% acetone, at last in acetone 3 times, embedded in Epon812 and polymerized at 35 °C for 16 h, then 48 °C for 24 h, and 65 °C for 48 h. For observation, ultrathin sections of the samples were cut with a diamond knife and collected on 200-mesh copper grids. After contrasting with uranyl acetate and lead citrate, the grids were examined with a JEM-2100 transmission electron microscope.

### Histochemical GUS staining

The histochemical GUS staining of homozygous T_3_ transgenic lines harbouring the *PDPG1*::*GUS* fusion gene was performed as previously described^57^. Images were recorded with a scanner (Epson perfection V30). At least 9 individual lines were analysed to produce the representative results shown here.

### Analysis of the chlorophyll contents

The chlorophyll content was analysed as described previously with minor modifications^58^. Fresh plant tissues were homogenized in 80% acetone, and debris were removed by centrifugation. The absorbance of the supernatant at 663 and 645 nm was measured with a 723B spectrophotometer (Tianjin PURUISI Equipment Co. LTD, China). All of the measurements were repeated in three independent experiments.

### Sequence alignment and phylogenetic analysis

The BLAST search program (http://blast.ncbi.nlm.nih.gov/) was used to search for the sequences of proteins homologous to AtDPG1. The multiple alignments were performed by using ClustalX 2.0 software^59^. To construct a phylogenetic tree, a clustal file was generated in the ClustalX 2.0 software using the protein sequences. The generated clustal file was downloaded and converted to MEGA file format using the MEGA5.1 software^60^. The generated MEGA file was then run in the MEGA5.1 software to construct the phylogenetic tree. The statistical parameters used to construct the phylogenetic tree were as follows: analysis: phylogenetic reconstruction, statistical method: Neighbor-joining, test of phylogeny: bootstrap method, no. of bootstrap replicates: 1000, substitution type: amino acids, model/methods: Jones-Taylor-Thornton (JTT) model, rates among sites: uniform rates, gaps/missing data treatment: partial deletion, site coverage cutoff: 95%.

## Additional Information

**How to cite this article**: Liu, D. *et al.* The novel protein DELAYED PALE-GREENING1 is required for early chloroplast biogenesis in *Arabidopsis thaliana.* *Sci. Rep.* **6**, 25742; doi: 10.1038/srep25742 (2016).

## Supplementary Material

Supplementary Information

## Figures and Tables

**Table 1:** Segregation of green and albino seeds in heterozygous *dpg1* mutant.

Total no. of seeds	Morphology		Null hypothesis	χ^2^	P
	Green	albino			
902	683	219	3:1	0.25	P > 0.05
