# Supplementary material for: The novel protein DELAYED PALE-GREENING1 is required for early chloroplast biogenesis in Arabidopsis thaliana
Source: Sci Rep. 2016 May 10;6:25742. doi: 10.1038/srep25742 (PMC4861969; doi:10.1038/srep25742)
Supplement: Supplementary Information [file srep25742-s1.pdf]

The novel protein DELAYED PALE-GREENING1 is required for early chloroplast biogenesis in *Arabidopsis thaliana*

Dong Liu*, Weichun Li & Jianfeng Cheng

College of Agronomy/Key Laboratory of Crop Physiology, Ecology and Genetic Breeding, Ministry of Education, Jiangxi Agricultural University, Nanchang, 330045, China

Author of correspondence:

Dong Liu

College of Agronomy/Key Laboratory of Crop Physiology, Ecology and Genetic Breeding, Ministry of Education

Jiangxi Agricultural University

Nanchang 330045

China

Tel: (86) 0791-83828081

Fax: (86) 0791-83828081

E-mail: liudongbio@163.com

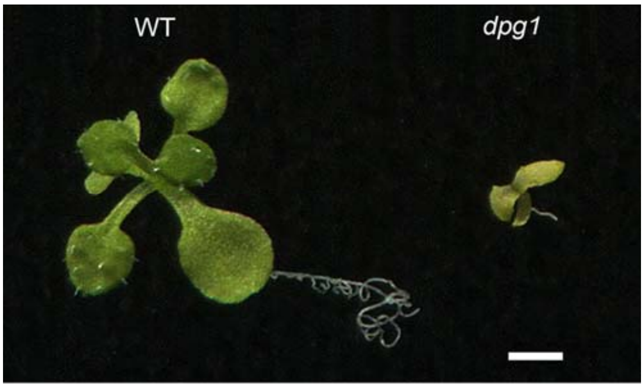

Supplementary Figure S1. Phenotypes of the wild type (WT) and *dpg1* mutant grown on 1/2 MS medium without sucrose for 28 days.

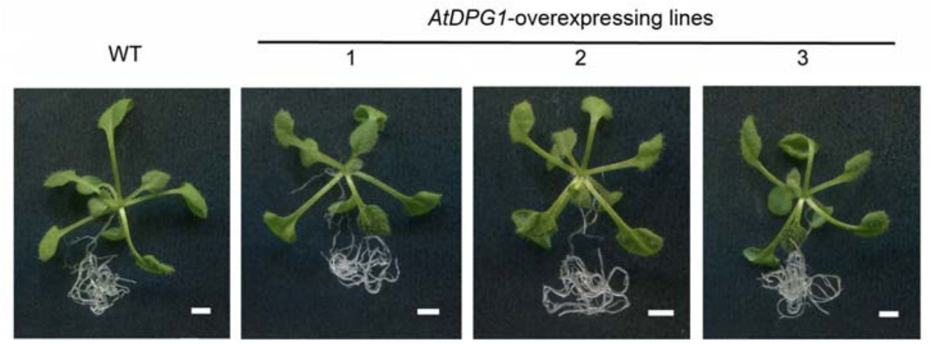

Supplementary Figure S2. Phenotypes of wild-type (WT) and *AtDPG1*-overexpressing lines grown on 1/2 MS medium for 21 days. Scale bar = 2 mm.

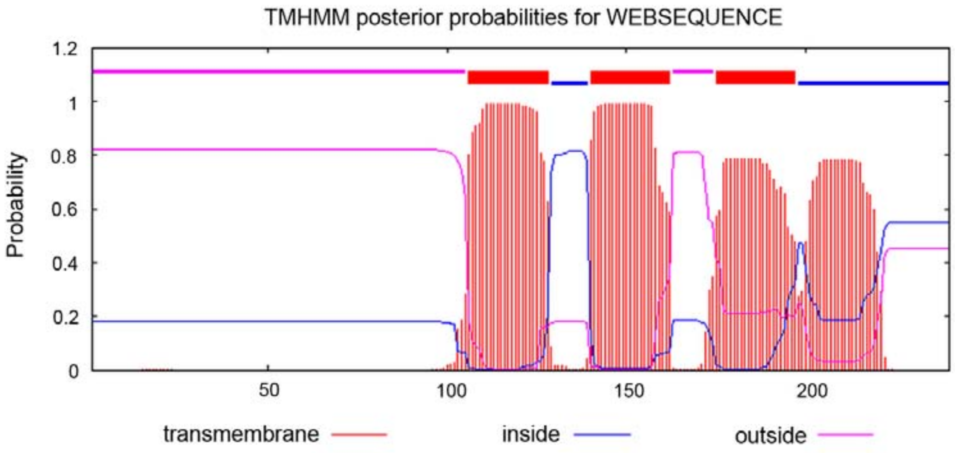

Supplementary Figure S3. Presence of transmembrane domain was calculated using TMHMM 2.0 Server. Red colour represents the transmembrane helices. The prediction indicates the presence of 3 domains in AtDPG1.

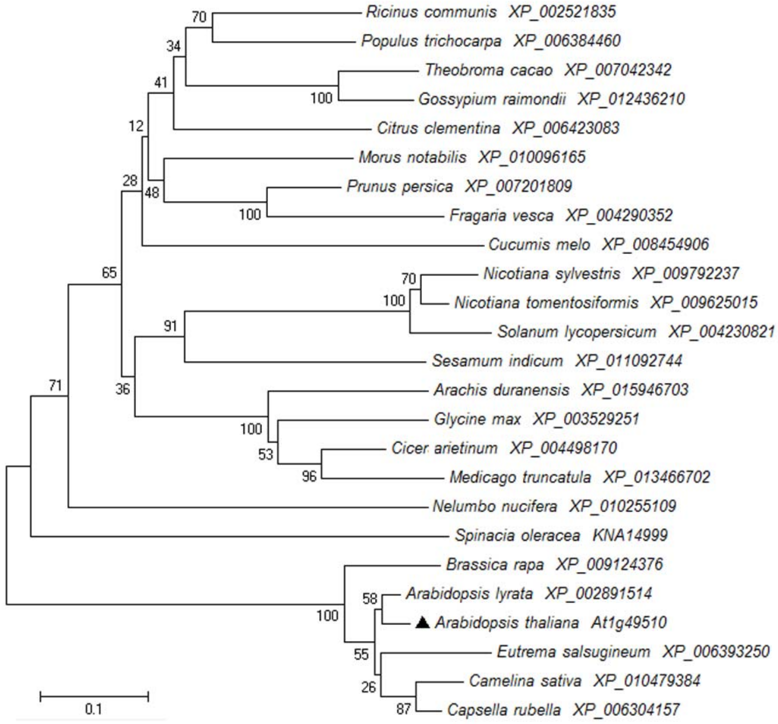

Supplementary Figure S4. A phylogenetic analysis of putative DPG1 homologues using MEGA 5.1 and a neighbor-joining method. A bootstrap analysis was performed with 1 000 replicates. Numbers in branches indicate bootstrap values (%).

Supplementary Table S1. Decreased fertility in the *dpg1* mutant

	WT	*dpg1*
Total number of siliques per plant	157.2±31.5	25.3±7.2*
Total number of seeds per silique	54.5±3.1	12.1±2.4*

Each value is the mean±SE of three independent determinations and asterisk indicates significant differences (Student's *t* test: *P＜0.05)

Supplementary Table S2. List of primers used in this study

Primer name	Primer sequence (5´-3´)	Function
LBb1.3	ATTTTGCCGATTTCGGAAC	
DPG1-LP	ACGAGTCCGACGTTGTGATAC	Identifying *dpg1* mutant by PCR
DPG1-RP	TCCCAAACATGTTTGGTTCTC	
PDPG1-F	CCCAAGCTTATTGGTAACTATCAAAGCG	Amplification of *AtDPG1*
PDPG1-R	CATGCCATGGTTATTTATTAAACTCTCTGT	promoter
OEDGY1-F	CATGCCATGGCGATGTCTCATCTCTTTCTT	Amplification of *AtDPG1* coding
OEDGY1-R	GATGGGTAACCTCATTCTGTTTCCAAACTG	region
AtDPG1-F1	ACGATTTCTCTCTCTCTCATTAGGC	Identifying *dpg1* mutant by
AtDPG1-R1	ACAATCATGGGTTGGTTTCTTACTT	semi-quantitative PCR
AtDPG1-F2	TCTCTTTCTTTCTTCTTCGCCTC	Identifying 35S::*AtDPG*1
AtDPG1-R2	CTTAAGACGAGGCGAGATCTTCA	transgenic lines by semi-quantitative PCR
ACTIN2-F	CAAACGAGGGCTGGAACAAGACT	Semi-quantitative PCR analysis
ACTIN2-R	GCAACTGGGATGATATGGAAAAGA	of *ACTIN2* gene
rAtDPG1-F	TCTCTTTCTTTCTTCTTCGCCTC	Quantitative PCR analysis of
rAtDPG1-R	TCCCAAACATGTTTGGTTCTC	*AtDPG1* gene
rPSAA-F	TCCTGAATGGAGATGTGGGCG	Quantitative PCR analysis of
rPSAA-R	TAAGGCTGCGAAGACCAATGC	*PSAA* gene
rPSAB-F	GTATTACCGCATCCCCAAGGG	Quantitative PCR analysis of
rPSAB-R	GGGTTAGAATGGCAGTTCCGG	*PSAB* gene
rPSBA-F	TGAATGGCTATACAACGGCGG	Quantitative PCR analysis of
rPSBA-R	GAAAACAGCAGTCGCAGCTGC	*PSBA* gene
rPSBB-F	CGTTGGGTATATTAGCGGGCC	Quantitative PCR analysis of
rPSBB-R	AGGACGGTTTCAATATTGCCC	*PSBB* gene
rRBCL-F	CCTGGAGTTCCACCTGAAGAAGC	Quantitative PCR analysis of
rRBCL-R	TGGTAGCATCGTCCTTTGTAACG	*RBCL* gene
rrpoA-F	ACGCGCTTATTTGTGTCCAAGG	Quantitative PCR analysis of
rrpoA -R	GACTATATCCGCGATTCCTCTC	*rpoA* gene
rrpoB-F	ATTGGATCATAATGGGATTTCG	Quantitative PCR analysis of
rrpoB -R	CTCGTAGATTCAAACCCATAGC	*rpoB* gene
rrpoC1-F	CTATAAAGAGTGGAATTTGCGC	Quantitative PCR analysis of
rrpoC1 -R	CCCATTTGATATCTTCGTATCC	*rpoC1* gene
rrpoC2-F	AACGATACCTTCTAAGGGCTGG	Quantitative PCR analysis of
rrpoC2 -R	ACCATATCTCAATAGATTGGCG	*rpoC2* gene
rHEMA1-F	TAATGGGGTTCGTGTTCTTCCG	Quantitative PCR analysis of
rHEMA1-R	ATGCTAGCTGCATTAGACGCAG	*HEMA1* gene
rCHLH-F	TGGTAGAGAGACAGAAGCTCGAAA	Quantitative PCR analysis of
rCHLH-R	CCAAAGAACCTGCCCAAGAG	*CHLH* gene
rCHL27-F	TCAAGACCGATTACAACCAGACA	Quantitative PCR analysis of
rCHL27-R	CGCTCAAGGAACTCAACGAAG	*CHL27* gene
rCHLP-F	ACCAGAAACAGAGCTAAGGACAAGA	Quantitative PCR analysis of

rCHLP-R	GCATCACCTACAAGAGCCACAC	*CHLP* gene
rPORB-F	CAAACCGCTGCGACTTCAAGC	Quantitative PCR analysis of
rPORB-R	TGCACGCCATTATCACGTTCC	*PORB* gene
rPORC-F	CAGACAGTTACAGCCACGCCG	Quantitative PCR analysis of
rPORC-R	TGTCTGCTAAAGCTTTGGCCG	*PORC* gene
rCAO-F	TAGGGGTGAAGACGGGAAACC	Quantitative PCR analysis of
rCAO-R	CTCCATCGGTTGAGTATTCCC	*CAO* gene
rCLA1-F	GGTGGAACCGGGTTAAATCTC	Quantitative PCR analysis of
rCLA1-R	TGCACAGAAGGGTTTAAGGCC	*CLA1* gene
rOE23-F	GTATCTCCTGCTGATGCCGCC	Quantitative PCR analysis of
rOE23-R	TGGAACCTGCACTTTGAACCC	*OE23* gene
rOE33-F	GTATCTCCTGCTGATGCCGCC	Quantitative PCR analysis of
rOE33-R	TGGAACCTGCACTTTGAACCC	*OE33* gene
rPSAN-F	CTGTGATCAAAGCTCAACGCG	Quantitative PCR analysis of
rPSAN-R	TTGGTTTTGCTCCTCTCGAGG	*PSAN* gene
rPSBW-F	TCTTGGTTTGCCTCCAATGGG	Quantitative PCR analysis of
rPSBW-R	CTCATCAACCAAAGCCATCGC	*PSBW* gene
rLHCB1-F	GGTTTGTGTTTGTGGTGGATGGTA	Quantitative PCR analysis of
rLHCB1-R	GTGAACCCAAGAACTGAAAATCCA	*LHCB1* gene
rLHCA4-F	AACCCGCTTAACTTTGCTCCTAC	Quantitative PCR analysis of
rLHCA4-R	CAAACCCTAAGAATGCCAACATC	*LHCA4* gene
rLHCB4-F	CAAGTTCTTTGACCCGCTAGG	Quantitative PCR analysis of
rLHCB4-R	GATGATGGTGGTGTGGAGTGG	*LHCB4* gene
rRBCS-F	GGTCGCTCCTTTCAACGGACTT	Quantitative PCR analysis of
rRBCS-R	ATTCGGAATCGGTAAGGTCAGG	*RBCS* gene
rGLK1-F	TATGACGGTGACAGTGACCGG	Quantitative PCR analysis of
rGLK1-R	AACTGTTCCACTGCCTCCACG	*AtGLK1* gene
rGLK2-F	TGTGTGTAAGCAAGAGGGTGG	Quantitative PCR analysis of
rGLK2-R	CTACCCCTAATTGCTCCACCG	*AtGLK2* gene
rACTIN2-F	CAAACGAGGGCTGGAACAAGACT	Quantitative PCR analysis of
rACTIN2-R	CTGTTGACTACGAGCAGGAGATGG	*ACTIN2* gene

Supplementary Table S3. The offspring segregated rates of the *dpg1* heterozygous carrying the *35S::AtDPG1* construction

Lines	Morphology		Segregated rates
	Green	albino	
1	185	7	26:1
2	176	17	10:1
3	210	14	15:1
4	169	17	10:1
5	113	23	5:1
6	39	3	13:1
7	68	2	34:1
8	37	6	6:1

Supplementary Table S4. The prediction of subcellular localization of plant DPG1 proteins

Locus number	Gene name	Number of amino acid residues	Prediction of the subcellular localization by ChloroP 1.1[a]			
			Score[b]	cTP[c]	CS-score[d]	cTP-length[f]
At1g49510	*AtDPG1*	240	0.561	Y	2.351	55
XP_002891514	*AlDPG1*	237	0.520	Y	4.081	36
XP_010479384	*CsDPG1*	237	0.520	Y	0.855	41
XP_006304157	*CrDPG1*	238	0.512	Y	2.351	54
XP_006393250	*EsDPG1*	239	0.575	Y	3.356	40
XP_009124376	*BrDPG1*	224	0.493	Y	1.581	26
XP_006423083	*CcDPG1*	249	0.519	Y	8.406	57
XP_007042342	*TcDPG1*	256	0.506	Y	4.303	49
XP_010096165	*MnDPG1*	247	0.538	Y	-0.817	54
XP_007201809	*PpDPG1*	237	0.499	-	4.207	25
XP_009792237	*NsDPG1*	253	0.568	Y	1.269	64
XP_006384460	*PtDPG1*	231	0.542	Y	0.318	19

[a]Subcellular localization predicted by the ChloroP 1.1 program (http://www.cbs.dtu.dk/services/ChloroP/). Score[b]: Output score from the second step network, the prediction cTP/no cTP is based solely on this score. cTP[c]: Whether or not this is predicted as a cTP-containing sequence; "Y" means that the sequence is predicted to contain a cTP; "-" means that is predicted not to contain a cTP. CS-score[d]: The MEME scoring matrix score for the suggested cleavage site. cTP-length[f]: The predicted length of the presequence (Please note that the prediction of the transit peptide length is carried out and presented even if its presence is not predicted). Similar results were obtained when using the PREDOTAR V1.03 (http://urgi.versailles.inra.fr/predotar/predotar.html) and IPSORT (http://hc.ims.u-tokyo.ac.jp/iPSORT/) subcellular localization tools.
